# Decreased variability of small-world propensity during non-response tasks in schizophrenia

**DOI:** 10.1016/j.ynirp.2026.100368

**Published:** 2026-06-18

**Authors:** Bryn Crandles, Michelle Jetha, Louis A. Schmidt, Sidney J. Segalowitz, William Marshall

**Affiliations:** a Department of Statistics and Actuarial Science, University of Waterloo, 200 University Ave W, Waterloo, Ontario, N2L 3G1, Canada; b Department of Psychology, Cape Breton University, 1250 Grand Lake Rd, Sydney, Nova Scotia, B1M 1A2, Canada; c Department of Psychology, Neuroscience & Behaviour, McMaster University, 1280 Main St W, Hamilton, Ontario, L8S 4L8, Canada; d Department of Psychology, Brock University, 1812 Sir Isaac Brock Way, St. Catharines, Ontario, L2S 3A1, Canada; e Department of Mathematics & Statistics, Brock University, 1812 Sir Isaac Brock Way, St. Catharines, Ontario, L2S 3A1, Canada

**Keywords:** EEG, Schizophrenia, Small-world

## Abstract

Functional disconnection is thought to underlie schizophrenia and is often studied by characterizing the small-worldness of networks derived from electroencephalogram (EEG) recordings. Decreased small-worldness in schizophrenia has been regularly observed during active tasks, while the results are varied for non-response or resting tasks. We obtained EEG networks in three non-response tasks from 42 (Mage = 42.07 years, SD = 6.38) individuals with schizophrenia and 42 matched controls and found that the schizophrenia group displayed reduced variation in small-worldness across tasks, measured using small-world propensity. We speculate that this observation can be attributed to aberrant functioning of the default mode network. Further, using ordinal regression, we showed that small-world propensity and its variation across tasks are associated with PANSS symptom scores.

## Highlights

• Small-world propensity (SWP) measured EEG network small-worldness in schizophrenia.
• Reduced variance in SWP values across non-response tasks in schizophrenia.
• SWP task values and variances across tasks predict PANSS symptom scores.

## 1 Introduction

Functional segregation is a well-studied principle of brain organization. Nerve cells with common functional properties are grouped together in densely interconnected clusters to perform specialized tasks (Friston 2002).While studying segregation has been largely successful, there are aspects of brain function that cannot be explained, for example, the existence of a unified cognitive moment (Varela et al., 2001). Functional integration has emerged as a complementary principle used to understand the interactions among specialized clusters, and how they contribute to various cognitive processes (Friston 2002). There is a natural tension between these two organizational principles – a fully segregated network that has no integration, versus a fully integrated network that has no segregation (Tononi, Sporns and Edelman, 1994). Studying the balance between functional segregation and function integration in the brain can further our understanding of how the brain performs various cognitive functions and may be of use to understand how these cognitive functions are disrupted in those affected by brain and mental health disorders.

The balance between functional segregation and functional integration is often studied in the context of small-world networks. Watts and Strogatz (1998) introduced the small-world network as an intermediary between a regular network and random network, which exhibits the highly clustered structure of a regular network while introducing some long-range connections. This structure facilitates effective synchronization and fast communication between nodes (Watts and Strogatz, 1998), and thus is a desirable architecture for a functional brain network to balance segregation and integration. Several metrics have been developed to quantify small-worldness, including the small-world index (SWI) (Humphries and Gurney, 2008), the measure “ω” in Telesford et al. (2011) and small-world propensity (SWP) (Muldoon et al., 2016), which incorporate a measure of clustering (the clustering coefficient) and a measure of path length (the characteristic path length: the number of edges traversed to get from one node to another). The clustering coefficient characterizes functional segregation, and integration is characterized by the characteristic path length. There is a large body of evidence demonstrating that both anatomical and functional human brain networks have small-world properties (Achard et al., 2006; Achard and Bullmore, 2007; Bassett and Bullmore, 2006, 2017; He et al., 2007; Salvador et al., 2005).

Schizophrenia is one area of particular interest for studying functional brain networks. It has been proposed that functional disconnection between brain regions is an underlying mechanism for the emergence of this disorder (Friston, 1998). Several theories have been proposed that further describe failure to properly integrate neural regions in schizophrenia (Andreasen et al., 1998, 1999; Peled, 1999; Touskova and Bob, 2015). This “disconnection hypothesis” has been empirically investigated in numerous studies. In an electroencephalogram (EEG) study on schizophrenia, clusters of neural activity were observed during a rest condition (Breakspear et al., 2003). In a functional magnetic resonance imaging (fMRI) study, reduced connectivity was observed in several sensorimotor networks at rest (Kaufmann et al., 2015). Evidence of disturbed sensory and cognitive integration was found using a structural equation model for task performance measures (de Jong et al., 2013; Ullman and Bentler, 2012). To investigate this disconnection hypothesis further, researchers have focused on studying the small-world architecture of functional brain networks, which serves as a measure of the balance between functional integration and segregation. The functional disconnection hypothesis predicts a higher clustering coefficient (higher segregation) and/or a lower characteristic path length (lower integration) in schizophrenia compared to a healthy brain. Such differences would impact the balance between segregation and integration and thus would be reflected in the small-worldness of the functional network.

Numerous studies have investigated the small-world architecture of brain networks in individuals diagnosed with schizophrenia. These studies differ in experimental paradigms, the measure of functional connectivity used, the type of network used (binary vs. weighted), and the nature of the task (actively responding vs. simply observing). Many have reported decreased small-world architecture in individuals with schizophrenia compared to healthy controls in networks derived from EEG data during active tasks that demand sustained and selective attention. For example, decreased small-world architecture has been observed during working memory tasks (Jhung et al., 2013; Micheloyannis et al., 2006; Pachou et al., 2008; Yao et al., 2021) and auditory oddball tasks (Shim et al., 2014). Mixed findings have been reported for resting conditions (no presented stimulus) and non-response tasks (stimulus presented but no response required) in EEG studies on both inpatient and outpatient samples (Gomez-Pilar et al., 2017; Jalili and Knyazeva, 2011; Jhung et al., 2013; Micheloyannis et al., 2006; Naim-Feil et al., 2018; Rodríguez-Lorenzana et al., 2021; Rubinov et al., 2009). Micheloyannis et al. (2006) and Rodríguez-Lorenzana (2021) observed decreased small-worldness for patients with schizophrenia during rest, while Gomez-Pilar et al. (2017) found that patients with schizophrenia had higher clustering and small-world properties in the baseline resting condition compared to controls. The authors attributed these results to the disconnection hypothesis, which supports the highly segregated networks observed in the patient group. Jhung et al. (2013) and Naim-Feil et al. (2018) reported no differences between groups in control/baseline tasks. Jalili and Knyazeva (2011) found differences in small-worldness (patients showed higher small-worldness in alpha EEG, but controls showed higher small-worldness in beta EEG) but found that the lack of differences in small-worldness depended on the network estimation procedure used. Rubinov et al. (2009) observed lower clustering and shorter path lengths in schizophrenia in a resting task, which the authors suggest may reflect greater randomization in their networks. In resting state functional magnetic resonance imaging (fMRI) studies (eyes closed), decreased small world architecture was observed in schizophrenia (Liu et al., 2008; Lynall et al., 2010). Thus, evidence points clearly toward altered small-world architecture in schizophrenia in active or executive tasks, while for resting conditions, the conclusion is unclear.

### 1.1 The present study

The current study was novel in that we used small-world propensity (SWP) (Muldoon et al., 2016) to quantify the small-worldness of the derived brain networks which, to our knowledge, has not been used in previous EEG studies on small-world architecture in schizophrenia. In contrast to SWI, SWP is less dependent on network density, and it more accurately characterizes the progression from a regular network to a small-world network to a random network (Muldoon et al., 2016). We investigated small-worldness in three experimental conditions: resting (eyes open and eyes closed), an auditory task where participants were asked to listen to music that varied in emotional content, and a visual task where participants were asked to view faces that varied in emotional expression. We found that mean small-worldness did not differ significantly between healthy controls and individuals with schizophrenia; yet, compared to healthy controls, patients with schizophrenia exhibited decreased variation in their SWP across tasks in all frequency bands. Finally, we showed that SWP values and variances across tasks are associated with Positive and Negative Syndrome Scale (PANSS) symptom scores (Kay et al., 1987) using an ordinal regression model.

## 2 Methods

### 2.1 Participants

Across analyses, participants were 42 outpatients (mean age = 42.07 years, SD = 6.38 years, range = [27, 56]; 69.04% male) with a formal diagnosis of schizophrenia spectrum disorders and 42 healthy controls (mean age = 39 years, SD = 8.08 years, range = [23, 55]; 66.66% male) who were matched on age and sex with the patient group. All patients attended the Hamilton Program for Schizophrenia (HPS), a community-based treatment and rehabilitation program (Whelton et al., 1999) in Hamilton, Ontario, Canada. All participants were stable outpatients and were living in the community (not in hospital) at the time of the research. Recruitment was conducted by an HPS research assistant who was familiar with the patients. Participants were contacted via phone and provided with detailed information about the study before being invited to take part. The exclusion criteria encompassed individuals with traumatic brain injury or concussions resulting in loss of consciousness, a history of learning disabilities, a reading level below grade 6, as well as those with chronic medical or neurological illness. Participants were closely monitored by their case managers, and no participants with any known substance use participated in the study. Diagnoses were carried out by a psychiatrist upon admission to the HPS program, following the DSM-IV-TR criteria (American Psychiatric Association, 2000). Diagnoses of schizophrenia and schizoaffective disorder were included in the study. Specifically, 26 of the 42 (62%) patients who participated in the study were diagnosed with paranoid schizophrenia, 8 (19%) with residual schizophrenia, 6 (14%) with schizoaffective disorder, 1 (2%) with disorganized schizophrenia, and 1 (2%) with undifferentiated schizophrenia. All patients were taking antipsychotic medications. Specifically, 33.33% were on clozapine, 28.57% on olanzapine, 21.43% on other atypical antipsychotics, and 16.67% on typical antipsychotics. On the day of testing, the psychiatric status of the participants was measured by the Positive and Negative Syndrome Scale (PANSS) (Kay et al., 1987). The PANSS is a widely used clinical assessment tool designed to evaluate the severity of symptoms in individuals with schizophrenia. It consists of 30 items that are categorized into three subscales: positive symptoms (e.g., hallucinations, delusions, grandiosity, thought disturbance, and hostility), negative symptoms (e.g., blunted affect, emotional and social withdrawal, lack of spontaneity, and stereotyped thinking), and general psychopathology (e.g., anxiety, depression, other non-psychotic symptoms). Higher scores in each subscale indicate greater psychopathology. Characteristics can be found in Table 1.

**Table 1:** Patient characteristics (n = 42). Mean (sd) presented for age and PANSS scores.

		PANSS Scores			
% Male	Age	Positive	Negative	General	Total
69.04%	42.07 (6.38)	12.55 (4.39)	12.19 (3.74)	26.07 (5.58)	50.81 (11.39)

Healthy controls were recruited from the metropolitan area of Hamilton, Ontario. Although a structured clinical interview was not administered to controls, when controls were initially contacted by phone for scheduling, they were queried regarding psychiatric status and history of psychiatric disorder. Individuals who reported current or prior history of psychiatric disorder were excluded from the study. Additional exclusion criteria included history of traumatic brain injury or concussions with loss of consciousness and neurological illness. When control participants arrived at the lab for testing, further questions were asked regarding medication status. Eleven controls reported taking common medications, including allergy, acid reflux, or thyroid medication. Controls did not report taking medications for psychiatric disorder, including antipsychotics, anti-depressants, or anxiolytics.

### 2.2 EEG procedures and measures

Participants were tested at the Child Emotion Laboratory at McMaster University. After providing written consent, participants were seated in an adjustable chair at a 1-m viewing distance from a computer screen. An experimenter was present at all times during the administration of instruments and was able to clarify any questions regarding any of the self-report measures. A trained clinician was present in the lab during all testing procedures with patients in the event that a participant became distressed. All procedures were approved by the McMaster and Hamilton Health Sciences Research Ethics Board. All participants received $20 remuneration for their participation. Electroencephalogram (EEG) was recorded using the 128-channel Geodesic Sensor Net (Electrical Geodesics, Inc.). Data were analog filtered 0.1 to 100 Hz, digitized at 250 samples per second, and referenced to the vertex (Cz). Sensor impedances were kept below 50 kΩ.

Three tasks were examined: resting (resting during eyes open and eyes closed conditions), listening to orchestral excerpts designed to induce emotions that varied in intensity and valence (i.e., joy, happiness, fear, and sadness), and viewing emotional faces without a response requirement (i.e., angry, fearful, happy, and neutral faces). Participants were asked to view the faces and to maintain a central fixation. Participants were not required to give an explicit response (e.g., with respect to the identification of emotion or gender) to ensure that task-related attentional demands would not alter the implicit processing of emotion. Participants were monitored from a separate room via a video monitor to ensure that they viewed stimuli during the task, to keep track of potential movement artifact, and to monitor the comfort level of participants. On average, the total task time plus inter-task time was 7 min for the resting condition, 5 min for the music condition, and 11 min for the passive viewing faces condition.

### 2.3 EEG reduction and analyses

The raw EEG was offline filtered 0.5 to 30 Hz and re-referenced to the average of all sites and then run through the EEG Integrated Platform Lossless (EEG-IP-L) pre-processing pipeline (Desjardins et al., 2021). This pipeline utilizes independent component analysis (ICA) to identify times and channels containing non-stationarity periods and noise (e.g., eye blinks, movement artifacts), and removes these from the analysis, with the remaining ICs identified as having a cortical origin (channels are removed for the entire session). The results were then projected back to the scalp and missing channels interpolated. Further examples of application and benefits of this pipeline are found in the literature (e.g., Henderson et al., 2024; van Noordt et al., 2020; van Noordt et al., 2015; Willoughby et al., 2021). In the Supplementary Materials (Table S1), we report pre-processing metrics, including average number of channels interpolated and proportion of ICs rejected by group. No evidence of differences in such metrics was found (number of channels: p = 0.86, proportion of ICs rejected: p = 0.22).

### 2.4 Coherence

Coherence is a frequency-based measure of functional connectivity. Given two signals $X_{j}\left(t\right)$ and $X_{k}\left(t\right)$, let $G_{jk}\left(f\right)$ be the cross spectral density at the frequency f, and let $G_{jj}\left(f\right)$ and $G_{kk}\left(f\right)$ be the marginal power spectral densities, respectively. The coherence between the two signals $X_{j}\left(t\right)$ and $X_{k}\left(t\right)$ is defined as

$$C_{jk}=\frac{{\left|G_{jk}\left(f\right)\right|}^{2}}{G_{jj}\left(f\right)G_{kk}\left(f\right)}\text{.}$$

The marginal power spectral density $G_{jj}\left(f\right)$ of signal $X_{j}\left(t\right)$ measures the power at a particular frequency f, while the cross spectral density $G_{jk}\left(f\right)$ measures the relation between $X_{j}\left(t\right)$ and $X_{k}\left(t\right)$ at that frequency. The coherence $C_{jk}$ quantifies the degree to which two signals’ oscillations are correlated over time (Nunez et al., 1997). In practice, we estimate the coherence by estimating $G_{jj}\left(f\right)$, $G_{kk}\left(f\right)$, and $G_{jk}\left(f\right)$ using the discrete Fourier transform of the observed data averaged across epochs. We first divide the signals into M 1 s overlapping epochs with an overlap of 500 ms. For each epoch m=1,…,M, we estimated the spectral densities G by first computing the Fourier transform

$$X_{j\left(m\right)}\left(f\right)=\sum_{t=1}^{T}X_{j\left(m\right)}\left(t\right)\exp\left(\frac{-i2\pi ft}{T}\;\right)$$

and then calculating the cross (power) spectral density between channels j,k, for j≠k (j=k):

$$\hat{G_{jk\left(m\right)}}\left(f\right)=X_{j\left(m\right)}\left(f\right)X_{k\left(m\right)}^{*}\left(f\right)$$

where $X_{j\left(m\right)}\left(f\right)$ is the Fourier transform of the $m^{th}$ epoch, and $X_{k\left(m\right)}^{*}\left(f\right)$ is the complex conjugate. We then averaged over the M epochs to obtain an overall estimate:

$$\hat{G_{jk}}=\frac{1}{M}\sum_{m=1}^{M}\hat{G_{jk\left(m\right)}}\left(f\right)$$

Prior to computing the DFT, we applied the Hann window function to each signal reduce spectral leakage. The DFT of the $m^{th}$ signal is then:

$$X_{j\left(m\right)}\left(f\right)=\sum_{t=1}^{T}X_{j\left(m\right)}\left(t\right)w\left(t\right)\exp\left(\frac{-i2\pi ft}{T}\right)$$

where w(t) is the window function.

### 2.5 Functional brain networks

Given the estimated pairwise coherence between electrodes, we estimated binary graphs representing the functional brain network. For a given frequency band, two electrodes *i* and *j* were connected by an edge if the sum of the coherence in the frequency band was greater than or equal to a given threshold. Otherwise, the electrodes were not connected. For each graph, the threshold was chosen such that the average degree (number of connections per electrode) was equal to ten. Binary graphs were estimated using a proportional threshold corresponding to ∼15.6% network density (an average degree of 10). We note that there is no consensus on using absolute or proportional thresholds in network analysis (Garrison et al., 2015; Masuda et al., 2025), and weighted networks can introduce additional noise into the analysis (Masuda et al., 2025). Garrison et al. (2015) found that network properties are more stable across proportional thresholds compared to absolute thresholds. Additionally, Muldoon et al. (2016) explains that the range of possible values for the clustering coefficient and path length reduces as density increases, indicating that small-worldness in high-density networks is not a well-defined property. While SWP for a particular network is normalized by the clustering and path length in regular and random networks with the same density, small-world structure is still constrained by density. Thus, to make SWP comparisons across networks, we use a proportional threshold to avoid any confounding due to network density.

### 2.6 Small-world analysis

A small-world network, introduced by Watts and Strogatz (1998), is a network whose structure facilitates information transfer between nodes. This structure is a combination of high clustering and short characteristic path length. A network is highly clustered if, on average, a particular node's connections are also connected to each other, and has short characteristic path length if the number of edges traversed to get from one node to another is small for all nodes in the network (Watts and Strogatz, 1998). We quantified the small-worldness of the binary graphs using small-world propensity (SWP), a measure that captures both the average clustering and characteristic path length of the network (Muldoon et al., 2016). We followed the methodology outlined by Muldoon et al. (2016) and used their publicly available code to perform the SWP calculations. The clustering coefficient (C) and path length (L) are defined as:

$$C=\frac{1}{N}\sum_{i=1}^{N}C_{i},L=\frac{1}{N\left(N-1\right)}\sum_{i\neq j}^{N\left(N-1\right)}S_{ij}$$

where $C_{i}$ is the fraction of existing edges between the k nodes connected to node i out of a total allowable edges $\frac{k\left(k-1\right)}{2}$, N is the total number of nodes, and $S_{ij}$ is the length of the shortest path between nodes i and j (a path is a sequence of edges).

A small-world graph has high C like a regular graph, and low L like a random graph. Each node of a regular graph is connected by an edge to its n closest neighbouring nodes, resulting in a highly clustered, “regular” structure (Watts and Strogatz, 1998). A random graph introduces long-range connections between nodes that, in a regular graph, would otherwise not be connected. For a particular graph of interest, the SWP involves normalizing the observed value of C to quantify its deviation from a regular graph, and normalizing L to quantify its deviation from a random graph:

$$\Delta_{C}=\frac{C_{reg}-C_{obs}}{C_{reg}-C_{rand}},\Delta_{L}=\frac{L_{obs}-L_{rand}}{L_{reg}-L_{rand}}$$

where the subscript obs corresponds to the observed graph, and where reg and rand correspond to a regular and random graph with the same number of nodes N and number of connections K as the observed graph. We calculated ($C_{reg},L_{reg}$) by generating a regular graph with N nodes that is connected to its $n=\lceil \frac{K}{2N}\rceil$ nearest neighbours and calculating the corresponding clustering coefficient and characteristic path length. We calculated $\left(C_{rand},L_{rand}\right)$ by generating a random graph with N nodes, and where the K edges in the original graph have been randomly redistributed.

As $C_{obs}$ increases, that is as the graph becomes highly clustered like a regular graph, $C_{reg}-C_{obs}$ approaches 0, and thus $\Delta_{C}$ also approaches 0. If $C_{obs}$ is small, $\Delta_{C}$ is close to 1. Similarly, if $L_{obs}$ is large, $\Delta_{L}$ is close to 1. As $L_{obs}$ decreases and displays short characteristic path length like a random graph, $\Delta_{L}$ approaches 0. It is possible that $C_{obs}>C_{reg}$ or $C_{obs}<C_{rand}$, and that $L_{obs}<L_{rand}$ or $L_{obs}>L_{reg}$. In such cases, it is possible that $\Delta_{C}$ and $\Delta_{L}$ fall outside of (0, 1). When this occurs, $\Delta_{C}$ and $\Delta_{L}$ are set to the appropriate boundary value (e.g., if $\Delta_{C}$ is observed to be less than 0, then it is set to 0), so that both measures are bounded between 0 and 1.

The small-world propensity is then defined as (Muldoon et al., 2016):

(1) $$\begin{array}{c} \text{SWP}=1-\sqrt{\frac{\Delta_{C}^{2}+\Delta_{L}^{2}}{2}}\text{,} \end{array}$$

which is bounded between 0 and 1. As explained above, graphs with $\Delta_{C}$ close to 0 and $\Delta_{L}$ close to 0 display high clustering like a regular graph, and low characteristic path length like a random graph, respectively. Thus, a network will have SWP close to 1 if its structure is small-world, where 1 is maximum small-worldness. Conversely, a network will have SWP close to 0 if its structure is not small-world; that is, if it has low C and high L.

In this study, we use a modified form of SWP. It is possible that randomly generating a single random and regular network will produce extreme values of clustering and path length, which could produce a skewed SWP value. To provide a more stable SWP estimate, we generated 100 regular networks and 100 random networks and calculated $\Delta_{C}$ and $\Delta_{L}$ using the averaged versions of $C_{reg}$, $C_{rand}$, $L_{reg}$, and $L_{rand}$ before calculating SWP in Equation (1).

### 2.7 Ordinal regression for PANSS scores

We used ordinal regression to model PANSS scores using the small-world values and between-task variances as covariates. Ordinal regression, which differs from linear regression in that the outcome is an ordered categorical variable rather than a continuous measure. Whereas linear regression assumes equal spacing between outcome values and predicts a numeric score, ordinal regression instead models the probability of the outcome falling at or below each category threshold, without assuming equal distances between categories. In addition, the two models rely on different assumptions: linear regression assumes normally distributed residuals with constant variance, while ordinal regression typically assumes proportional odds across thresholds.

The proportional odds ordinal regression model assumes that the observed score for each individual i, denoted $Y_{i}$, has J observed levels. For each individual and each level j,j=1,…,J−1, the log odds ratio is modeled as a linear combination of the covariates,

$$\log\frac{P\left(Y\leq j\right)}{1-P\left(Y\leq j\right)}=\theta_{j}{-\beta }^{⊺}x_{i}\text{,}$$

where $P\left(Y_{i}\leq j\right)$ is the probability that individual i has score less than or equal to j given their covariates $x_{i}={\left(x_{i1},\ldots ,x_{ip}\right)}^{⊺}$ and $\beta^{⊺}=\left(\beta_{1},\ldots ,\beta_{p}\right)$ is the coefficient vector. This model is called the proportional odds model since the odds ratio given two values of covariates $x_{1}$ and $x_{2}$ is given by:

$$OR=\frac{\exp\left(\theta_{j}+\beta^{⊺}x_{1}\right)}{\exp\left(\theta_{j}+\beta^{⊺}x_{2}\right)}=\exp\left(\beta^{⊺}\left(x_{2}-x_{1}\right)\right)\text{,}$$

and so, the odds ratio is independent of the level j and proportional to the difference in covariate values. The exponentiated coefficient $\exp\left(\beta_{k}\right),k=1,\ldots ,p$ is the odds ratio of the event Y>j when $k^{th}$ covariate increases by one unit, and so positive coefficients indicate that larger covariate values increase the probability of larger scores.

To identify important covariates, we used bidirectional stepwise selection with Bayesian Information Criterion (BIC) as the criterion. This method starts with a model with no covariates, and at each step, one variable is added or removed, whichever minimizes the BIC:

klog(n)−2log(L),

where L denotes the likelihood, k is the number of parameters in the model, and n is the sample size.

## 3 Results

We computed the functional networks described in Section 2.4 for each participant and for each task in the delta (1-3 Hz), theta (4-7 Hz), alpha (8-12 Hz), and beta (13-30 Hz) frequency bands. The primary result of the paper is a significant difference between groups in the across-task variance of SWP (presented in Section 3.2). In the remaining sections, we investigate the source of the difference in variability and its relationship to PANSS scores.

### 3.1 Mean SWP

**Fig. 1:**
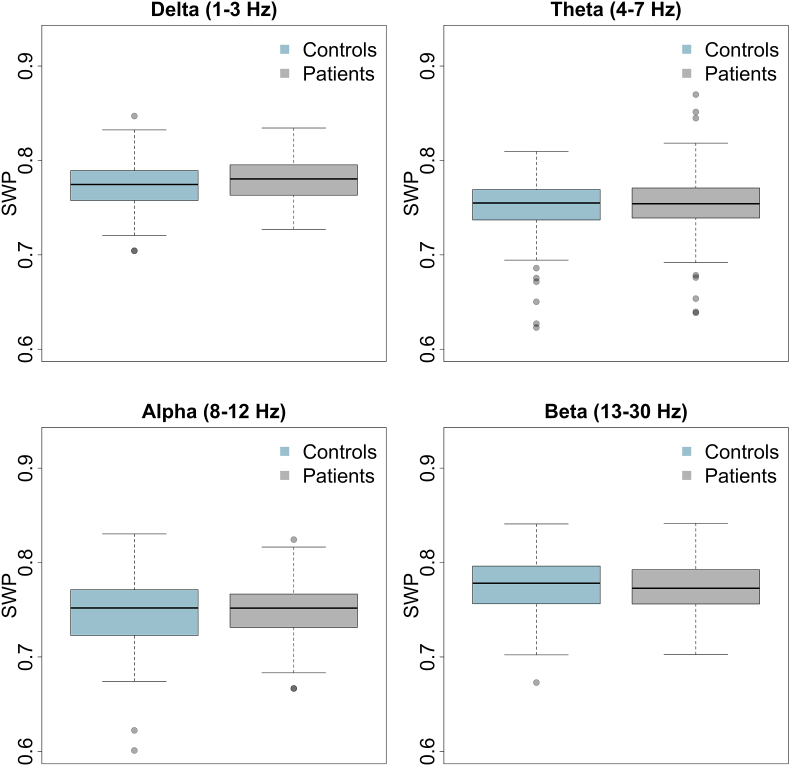
Box plots of SWP values for each frequency band. Delta is shown in the top left, where the small effect size is visualized (η^2^ = 0.0481).

We first compared the mean SWP between groups for each of the four frequency bands. A mixed effects ANOVA was performed with the group (control/patients) as the between-subjects factor, and the task (resting, music, faces) as the within-subjects factor. A group difference in SWP was found in the delta frequency band (F(1, 82) = 4.146, p = 0.045, $\eta^{2}$ = 0.0481), with a higher patient mean than control mean. No significant group differences were found in any of the other frequency bands (theta: p = 0.654, alpha: p = 0.793, beta: p = 0.723). The small effect size of the result in the delta band can be seen in the box plot of the SWP values in Fig. 1. When the potential outliers shown in Fig. 1 were removed from the analysis, we observed no change in the conclusions, i.e., there were no group differences found in SWP (delta: p = 0.057, theta: p = 0.737, alpha: p = 0.988, beta: p = 0.479). Based on the marginal effect size observed in delta, we concluded that the significant group effect was not interesting enough to warrant further investigation.

### 3.2 Variance of SWP

**Fig. 2:**
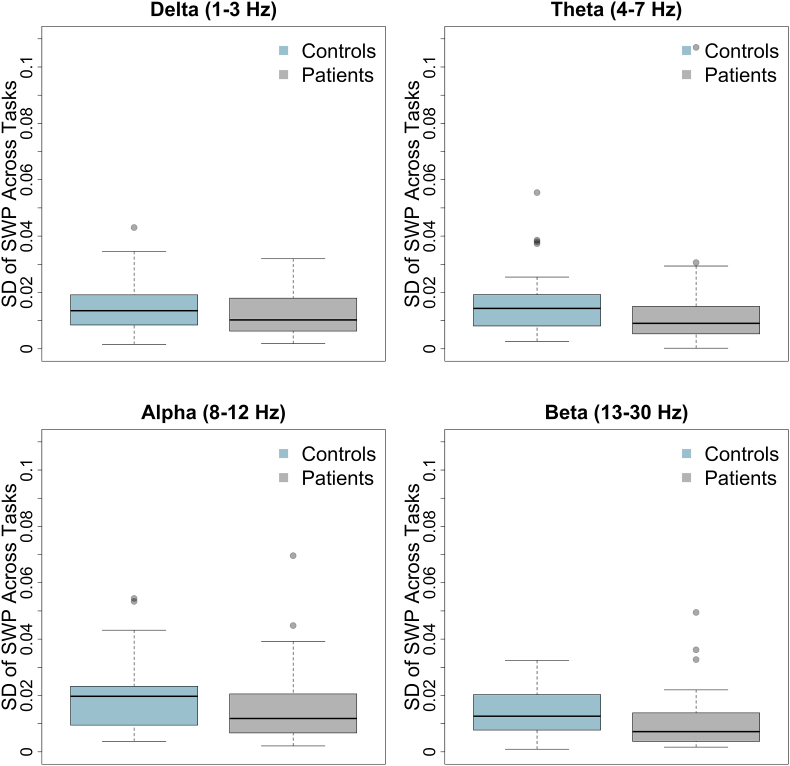
Box plots of SWP standard deviation across tasks. In each frequency band, controls demonstrate higher variability across tasks than patients (delta (1-3 Hz): p = 0.089, theta (4-7 Hz): p = 0.018, alpha (8-12 Hz): p = 0.043, beta (13-30 Hz): p = 0.003).

Next, for each participant, we computed the variance of SWP across tasks and then compared the variability across groups using the non-parametric Wilcoxon-Mann-Whitney rank sum test (Mann and Whitney, 1947; Wilcoxon, 1945) to identify differences in distributions/medians between groups. We found that the control group demonstrated higher variability in SWP across tasks compared to the patient group in all frequency bands, as shown in Table 2. We applied both Bonferroni (Dunn, 1961) and Benjamini-Hochberg (Benjamini and Hochberg, 1995) corrections for multiple comparisons. The Benjamini-Hochberg correction is less conservative and may be more appropriate under dependence. There is evidence of a significant difference (p < 0.05) or a trend (p < 0.1) in all frequency bands with the Benjamini-Hochberg procedure. Boxplots of the SWP standard deviation across tasks are shown in Fig. 2.

**Table 2:** Mann-Whitney asymptotic rank tests on differences in SWP standard deviation across tasks with Cohen's d as a measure of effect size: d = T/√N, where N = 84 is the total sample size, as well as Bonferroni and Benjamini-Hochberg corrections.

	T	p-value	Bonferroni p-value	Benjamini-Hochberg p-value	Effect size
Delta (1-3 Hz)	1.700	0.089	0.357	0.089	0.185
Theta (4-7 Hz)	2.371	0.018	0.071	0.036	0.259
Alpha (8-12 Hz)	2.022	0.043	0.173	0.058	0.221
Beta (13-30 Hz)	2.925	0.003	0.014	0.014	0.319

We investigated the sensitivity of these results to (1) outliers and (2) the proportional threshold chosen. The results are robust when the potential outliers indicated in Fig. 2 are removed. In delta, one outlier (control) was removed, in theta, six outliers (four controls and two patients) were removed, in alpha, four outliers (two controls and two patients) were removed and finally in beta, three outliers were removed (all patients). Table S2 of the Supplement provides results of the Mann-Whitney tests with outliers removed. The results are in general similar; however, in the delta frequency band, the evidence is slightly weaker (p = 0.126). Additionally, in Table S3 of the Supplementary Materials, we provide results of the Mann-Whitney tests for differences in SWP variance using other proportional thresholds (average degrees 8, 9, 11, 12). The result for the alpha frequency band is robust across thresholds. For both delta and theta bands, the result holds for thresholds 11-12, while for beta, the result also holds for threshold 12.

#### 3.2.1 Clustering and path length

To investigate which aspect of small-worldness (clustering or path length) drives the group difference in overall SWP variance, the variances in the clustering coefficient (C) and characteristic path length (L) across tasks were also examined using the Wilcoxon-Mann-Whitney test, with both Bonferroni and Benjamini-Hochberg corrections. In the theta and alpha frequency bands, we found that both C and L demonstrate a significant (or nearly significant) increase in variability across tasks in controls relative to patients. Conversely, in the delta and beta frequency bands, neither C nor L show such differences (Table 3). This suggests that neither parameter solely leads to the observed differences in SWP variance across tasks, but rather that a combination of variation in C and L results in the group differences for all frequency bands.

**Table 3:** Mann-Whitney asymptotic rank tests on differences in C and L standard deviation across tasks with Cohen's d as a measure of effect size d = T $/\sqrt{N}$ where N=84 is the total sample size, as well as Bonferroni and Benjamini-Hochberg corrections.

	Delta		Theta		Alpha		Beta	
	C	L	C	L	C	L	C	L
**T-statistic**	1.127	1.592	1.807	2.281	3.507	2.040	1.807	0.483
**p-value**	0.260	0.111	0.071	0.023	0.0005	0.041	0.071	0.629
**Bonferroni p-value**	1	0.445	0.283	0.090	0.002	0.166	0.283	1
**Benjamini-Hochberg p-value**	0.260	0.148	0.094	0.083	0.002	0.083	0.094	0.629
**Effect size**	0.123	0.174	0.197	0.249	0.383	0.223	0.197	0.053

#### 3.2.2 Variance of SWP in the clozapine subgroup

Another consideration is that the schizophrenia group comprised stable outpatients receiving antipsychotic medications. Antipsychotic medications have been shown to alter functional brain activity over the course of treatment (Davis et al., 2005), and their effects vary considerably across individuals (Lieberman et al., 2005; Strous et al., 2004). Clozapine, in particular, has been associated with pronounced neurophysiological effects. EEG slowing appears more frequent with clozapine than with other antipsychotics (Jackson and Seneviratne, 2019), and it has also been linked to alterations in connectivity patterns (Blazer et al., 2022) and coherence (Knott et al., 2002). Such effects may influence network organization by altering patterns of neural activity, with potential implications for the flexibility of network dynamics across task contexts.

**Fig. 3:**
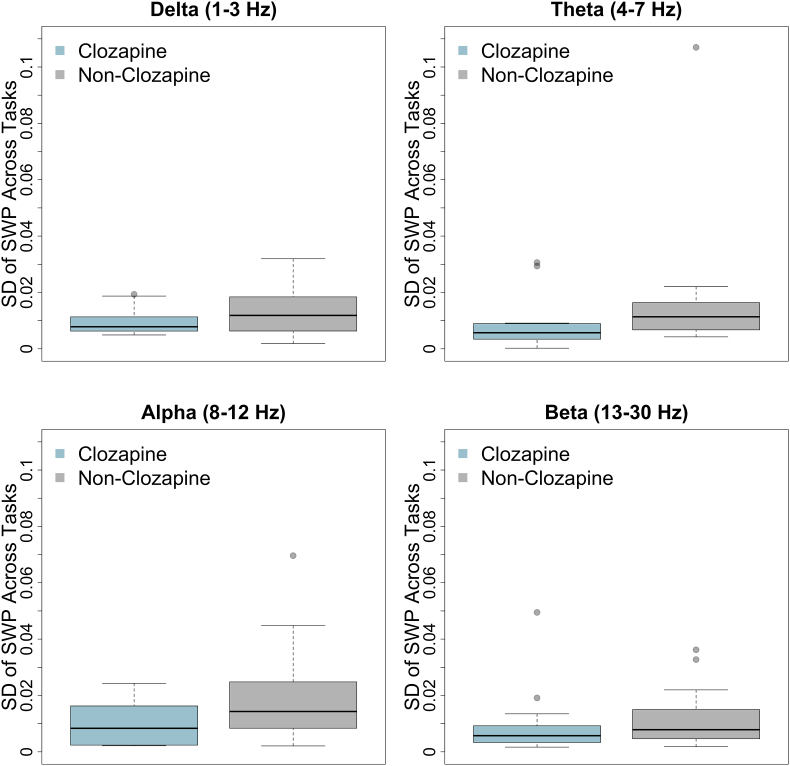
Box plots of SWP standard deviation across tasks for patients on clozapine and on non-clozapine medications.

Given that approximately one-third of our sample was prescribed clozapine, we examined whether SWP variability differed between patients receiving clozapine and those on other antipsychotic medications. We divided the patients into those on clozapine (n = 14) and those on non-clozapine medications (n = 28) and performed the Wilcoxon-Mann-Whitney test for differences in SWP variance. We observed some evidence of reduced variability in the clozapine group versus the non-clozapine group in theta and alpha frequency bands (see Table 4 and Fig. 3).

**Table 4:** Wilcoxon-Mann-Whitney asymptotic rank tests on differences in SWP standard deviation across tasks in the clozapine versus non-clozapine subgroups, with Cohen's d as a measure of effect size: d = T/√N, where N = 42 is the total sample size, as well as Bonferroni and Benjamini-Hochberg corrections.

	T	p-value	Bonferroni p-value	Benjamini-Hochberg p-value	Effect size
**Delta (1**–**3 Hz)**	−1.041	0.298	1	0.298	0.161
**Theta (4**–**7 Hz)**	−2.428	0.015	0.061	0.061	0.375
**Alpha (8**–**12 Hz)**	−1.814	0.070	0.278	0.139	0.280
**Beta (13**–**30 Hz)**	−1.067	0.286	1	0.298	0.165

#### 3.2.3 Localization

**Fig. 4:**
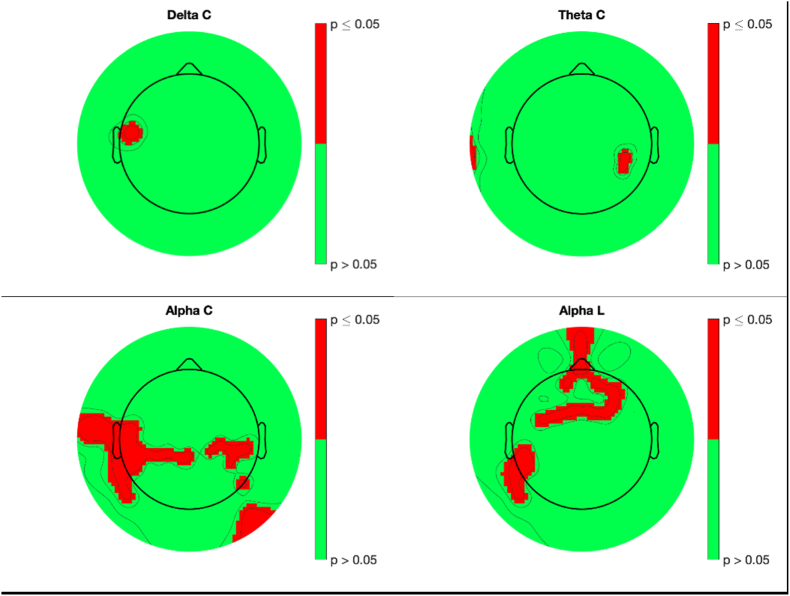
TFCE topographic maps demonstrating localized differences in variation across tasks with higher variance in controls compared to patients in delta C (top left), theta C (top right), alpha C (bottom left), and alpha L (bottom right).

*F*urther, both C and L (but not SWP) can be computed for individual nodes of the graph (individual electrodes). We used these individual node quantities to see if task variability can be isolated to specific regions. We used threshold-free cluster-enhancement (TFCE), a non-parametric method that corrects for multiple comparisons (Mensen and Khatami, 2013) to identify these regions. Topographic maps of the results are shown in Fig. 4. There were small regions of significant differences in C variability across tasks in delta (top left) and theta (top right). There were large clusters of differences in C and L variability in alpha (bottom left and right respectively). There were no significant differences for the other combinations (not shown).

#### 3.2.4 SWP heterogeneity

A simple premise for the increased SWP variability observed in controls is the following: patients with schizophrenia exhibit a constant SWP value across all tasks, whereas controls systematically exhibit lower SWP in one task compared to the others (e.g., lower SWP in resting compared to music and faces). This pattern would ultimately result in a group-task interaction effect on the mean SWP, i.e., the effect of task on SWP is different across groups (in this hypothetical case, the effect of resting is lower in patients compared to controls). However, no such interaction was observed in any frequency band (delta: p = 0.979, theta: p = 0.143, alpha: p = 0.778, beta: p = 0.257). Thus, there is likely heterogeneity in the source of SWP variance across tasks. We compared the heterogeneity in the control and patient groups using a statistic based on pairwise differences in SWP between tasks. For each group, and for each pair of tasks (i.e., resting/music, resting/faces, music/faces), we computed the following statistic:

$$R_{\left(i\right)}=\frac{\left|\bar{d_{\left(i\right)}}\right|}{\bar{d_{abs\left(i\right)}}}$$

for *i = *1,2,3*,* where *i* indicates the task pair, and where:

$$\bar{d_{\left(i\right)}}=\frac{1}{n}\sum_{j=1}^{n}{\text{SWP}}_{diff\left(i\right)}$$

$$\bar{d_{abs}}=\frac{1}{n}\sum_{j=1}^{n}\left|{\text{SWP}}_{diff\left(i\right)}\right|$$

and where ${\text{SWP}}_{diff\left(i\right)}$ is the task difference in SWP for each of the three task pairs.

A ratio $R_{\left(i\right)}$ close to 0 for a particular group and task pair indicates that individual subject task differences are heterogeneous within the group, while a ratio close to 1 indicates that individual subject task differences are homogeneous within the group. In Table 5, we see that although some band-pair comparisons show large differences in heterogeneity scores, the average scores across comparisons are quite similar (overall mean is 0.23 for control, 0.24 for patient). Thus, the degree of heterogeneity is similar in both groups, i.e., variance differences cannot be attributed to heterogeneity in one group versus homogeneity in the other.

**Table 5:** Ratios R describing the heterogeneity within the control and patient group separately, for each task pair.

	Task Pair	Controls Ratio	Patients Ratio
**Delta**	resting – music	0.156	0.75
	resting – faces	0.152	0.261
	music – faces	0.000	0.074
**Theta**	resting – music	0.392	0.604
	resting – faces	0.076	0.505
	music – faces	0.528	0.023
**Alpha**	resting – music	0.163	0.156
	resting – faces	0.025	0.206
	music – faces	0.190	0.022
**Beta**	resting – music	0.539	0.140
	resting – faces	0.319	0.114
	music – faces	0.221	0.025
**Overall Mean**		0.23	0.24

### 3.3 Clinical measures

Additionally, we associated PANSS scores in patients to SWP and SWP variance using ordinal regression. We used bidirectional stepwise selection with BIC as the criteria as described in Section 2.6, and evaluated model fit using likelihood ratio tests and pseudo-$R^{2}$ (defined as $1-l_{m}/l_{0}$, where $l_{m}$ is the log likelihood of the fitted model, and $l_{0}$ is the log likelihood of the null model) (McFadden, 1974). The results suggest that the predictors are associated with differences in PANSS scores, as shown in Table 6. The selected coefficients are shown in Table 7, Table 8, Table 9.

**Table 6:** Pseudo-$R^{2}$ and likelihood ratio test p-values for each of the ordinal regression models.

Clinical Measure	Pseudo-$R^{2}$	Degrees of freedom	LR test p-value
PANSS- Positive	0.130	6	0.0002
PANSS- Negative	0.029	1	0.018
PANSS- General	0.054	4	0.016

**Table 7:** Positive scores ordinal regression model selected using BIC.

Variable	Coefficient	Std. Error	p-value
Delta resting SWP	1.850	0.444	0.00003
Delta SWP variance	−1.184	0.393	0.003
Alpha music SWP	−1.286	0.486	0.008
Alpha SWP variance	−1.440	0.516	0.005
Beta resting SWP	−0.965	0.401	0.016
Beta SWP variance	0.566	0.273	0.038

**Table 8:** Negative scores ordinal regression model selected using BIC.

Variable	Coefficient	Std. Error	p-value
Alpha faces SWP	−0.6807	0.2744	0.013

**Table 9:** General scores ordinal regression model selected using BIC.

Variable	Coefficient	Std. Error	p-value
Alpha SWP variance	−1.069	0.358	0.003
Beta resting SWP	−1.832	0.560	0.001
Beta faces SWP	1.407	0.501	0.005
Beta SWP variance	0.757	0.343	0.0273

## 4 Discussion

In this work, we investigated properties of functional brain networks estimated from EEG recordings during three non-response tasks (resting, auditory perception, visual perception) in a sample of stable outpatient adults with schizophrenia and demographically matched healthy adults. Specifically, we calculated SWP (Muldoon et al., 2016) to quantify how closely the networks resembled a small-world architecture (Watts and Strogatz, 1998). We found no significant difference in the mean SWP across groups; however, individuals with schizophrenia exhibited reduced SWP variability across task conditions. We were able to link the differences in functional brain networks to clinical/behavioural outcomes by associating SWP values and SWP variance across tasks to the symptom scores of the patient group.

### 4.1 Default mode network

The default mode network (DMN) is a term used to describe the functional connectivity of the brain during “rest” (i.e., off-task time when there is no specific external task being performed). Investigation using fMRI and positron emission tomography have identified a collection of brain areas that consistently show functional connection during such rest and are collectively known as the DMN (including medial and lateral parietal, medial prefrontal, and medial and lateral temporal cortices) (Rachlie et al., 2015). The DMN has been extensively studied in schizophrenia (Hu et al., 2017), and decreased small-worldness has been observed during active tasks (Jhung et al., 2013; Micheloyannis et al., 2006; Pachou et al., 2008; Shim et al., 2014; Yao et al., 2021). In active tasks, reduced functional connectivity between the DMN and other functional networks was observed in patients with schizophrenia. Examples include reduced connectivity with the external attention system (EAS) during a working memory task (Pu et al., 2016) and with the dorsal attention, frontoparietal and cingulo-opercular networks during working memory (Godwin et al., 2017). This observed reduced connectivity between the DMN and other networks may explain differences in small-worldness in active tasks.

Differences in small-worldness between individuals with schizophrenia and healthy controls during resting conditions or in non-response tasks are less clear, with some studies showing decreased small-worldness (Micheloyannis et al., 2006; Rodríguez-Lorenzana et al., 2021), others showing increased small-worldness (Gomez-Pilar et al., 2017), and others no differences (Jhung et al., 2013; Naim-Feil et al., 2018). Acknowledging that spatial limitations in EEG recordings prevent direct measurement of DMN activity, our results provide some indirect evidence for no difference in the average small-worldness during resting conditions and during nonresponse tasks with low performance demands. We propose that aberrant function of the DMN can explain the decreased variability of EEG-based small-worldness observed during rest and non-response tasks. The proposed explanation cannot be confirmed based on the current data but could be explored in future work using simultaneous EEG-fMRI recordings.

Modelling studies have demonstrated that neural noise (random fluctuations neural activity) must remain within a specific range to support DMN functioning (Deco et al., 2009; Ghosh et al., 2008). Such neural noise is observable as trial-to-trial variability at the level of single cells to behaviour (Faisal et al., 2008) and in network dynamics (Deco et al., 2009; Ghosh et al., 2008). This variability should then also be observed in the estimated functional networks and their properties, including small-worldness. Thus, changes in the variability of the estimated functional networks (and thus of small-worldness) may be indicative of aberrant DMN functioning. Recent fMRI studies provide converging evidence for this interpretation, reporting reduced BOLD signal variability in DMN (Wei et al., 2023; Li et al., 2020) and reduced dynamic state transitions (state variability) among DMN states Kottaram et al., 2019) in patients with schizophrenia. Together, these findings indicate potential aberrant DMN functioning during rest and non-response tasks.

Moreover, in accordance with our results, aberrant DMN functioning (i.e., more frequent state transitions, reduced functional connectivity) is correlated with symptom scores (Weber et al., 2020). However, previously reported results often correlate symptom scores to specific changes in the local strength of functional connectivity between pairs of regions (e.g., negative symptom scores correlated with functional connection between right lateral parietal cortex and left temporal pole (Sasabayashi et al., 2023)), whereas the current results relate symptom scores to global functional network properties.

### 4.2 Attention

A complementary interpretation of our results is within the context of attention. No explicit behavioural or self-report data on attention were collected, but in a previous analysis of our data, the patient group showed a reduced N170 ERP amplitude compared to the control group in response to the face stimuli (Jetha et al., 2013) which could indicate reduced attention (although there are other potential interpretations, such as impaired face processing). It could be that the patient group is simply less attentive to the audio or visual aspects of the simple attention tasks than the control group is, leading to decreased variance across conditions. Indeed, a reduced effect in the startle reflex task indicates decreased passive attention in patients with schizophrenia (Dawson et al., 1993). Moreover, the magnitude of the reduction correlates with positive symptom scores (Dawson et al., 2000).

If the patient group is less attentive to the stimuli (e.g., music and faces), are they mind-wandering or attending to an internal stimulus? Mind wandering has been related to increased activity in the DMN in healthy participants (Kucyi, 2018; Mason et al., 2007; Scheibner et al., 2017). On the other hand, focused internal attention is related to reduced activity in the DMN relative to mind wandering and external attention (Scheibner et al., 2017), which would be consistent with the possibility of aberrant DMN functioning described above. Mind wandering may have more easily occurred during the resting task, given the absence of external stimulation. The music and face tasks provided auditory and visual input, and participants were monitored via video to ensure they faced the screen with eyes open; however, reduced attention to external stimuli may nevertheless have occurred in the absence of an active response requirement. In contrast, active tasks that require a behavioral response (e.g., a button press) provide trial-by-trial confirmation of engagement, thereby reducing the likelihood of mind wandering.

### 4.3 Strengths, limitations, and future directions

Compared to other small-world EEG studies in schizophrenia, the patient sample size (n = 42) used in this study was large (mean = 27.2, sd = 9.08, range = (14, 40) in EEG studies cited here). Further, potential confounding due to age and sex was controlled by matching patients to healthy controls. The small-world propensity used in this work is the most recently developed small-world measure that, to our knowledge, most accurately quantifies small-worldness as demonstrated by Fig. 1 in Muldoon et al. (2016).Averaging over many randomly generated networks was used to further enhance the stability of the SWP estimates. Compared to SWI, SWP is a (0, 1) measure, with 1 representing maximal small-worldness and is also less affected by network density (Muldoon et al., 2016). Studies on small-world architecture in schizophrenia vary in terms of the tasks investigated (e.g., resting, watching without responding, or active response tasks), the measure of functional connectivity, the use of binary vs. weighted graphs, and how small-worldness is quantified. We note that altering the choices made at various stages of the methodology (i.e., the use of coherence, binary graphs with a proportional threshold, and small-world propensity) may affect the conclusions drawn. However, we found that the variance result, particularly in alpha, is robust to the choice of proportional threshold.

Although the current study was not designed to directly assess medication-specific effects, we observed some evidence that decreased SWP variance is more pronounced in the clozapine subgroup. Future work could examine whether SWP variation differs as a function of medication type (e.g., clozapine versus other antipsychotics) or dosage. Such analyses would help clarify the extent to which the present findings reflect underlying pathophysiology versus treatment-related modulation of brain networks.

Future research would include further exploring the reduced SWP variation observed in schizophrenia, i.e., determining the mechanisms that result in this observation. This may also involve investigating if the current findings hold when temporal variability within tasks is computed. An alternative avenue of interest is to determine the subnetworks that maximize small-world propensity and investigate differences in SWP as well as regions included in the networks.

## Funding

This research was funded by 10.13039/501100000038NSERC operating grants to Louis Schmidt [grant number 203710-2011 and 203710-2006], an 10.13039/501100000038NSERC Discovery grant to William Marshall [RGPIN-2019-05418] and an 10.13039/501100000038NSERC doctoral award to Michelle Jetha.

## CRediT authorship contribution statement

**Bryn Crandles:** Formal analysis, Methodology, Software, Visualization, Writing – original draft. **Michelle Jetha:** Conceptualization, Investigation, Writing – original draft, Writing – review & editing. **Louis A. Schmidt:** Conceptualization, Investigation, Writing – review & editing. **Sidney J. Segalowitz:** Conceptualization, Investigation, Methodology, Supervision, Writing – original draft. **William Marshall:** Conceptualization, Methodology, Supervision, Writing – original draft.

## Declaration of competing interest

The authors declare that they have no known competing financial interests or personal relationships that could have appeared to influence the work reported in this paper.

## Data Availability

Data will be made available on request.
