# Supplementary material for: Decreased variability of small-world propensity during non-response tasks in schizophrenia
Source: Neuroimage Rep. 2026 Jun 18;6(3):100368. doi: 10.1016/j.ynirp.2026.100368 (PMC13311832; doi:10.1016/j.ynirp.2026.100368)
Supplement: Multimedia component 1 [file mmc1.docx]

*Table S1: EEG-IP-L pre-processing metrics. There was no evidence of group differences in mean number of channels removed using a t-test (p = 0.86), and no evidence of group differences in proportion of ICs rejected (p = 0.22).*

|  | **Controls** | **Patients** |
| --- | --- | --- |
| **Mean (SD) No. Channels Removed** | 14.8 (4.5) | 14.3 (4.4) |
| **Mean (SD) Prop. ICs Rejected** | 0.66 (0.13) | 0.63 (0.11) |

*Table S2: Mann-Whitney asymptotic rank tests on differences in SWP standard deviation across tasks with Cohen’s d as a measure of effect size: d = T/√N, where N=84 is the total sample size, as well as Bonferroni and Benjamini-Hochberg corrections, with outliers removed.*

|  | **T** | **p-value** | **Bonferroni  p-value** | **Benjamini-Hochberg p-value** | **Effect size** |
| --- | --- | --- | --- | --- | --- |
| **Delta (1-3 Hz)** | 1.530 | 0.126 | 0.504 | 0.126 | 0.168 |
| **Theta (4-7 Hz)** | 2.219 | 0.026 | 0.106 | 0.048 | 0.251 |
| **Alpha (8-12 Hz)** | 2.098 | 0.036 | 0.144 | 0.048 | 0.235 |
| **Beta (13-30 Hz)** | 3.639 | 0.000 | 0.001 | 0.001 | 0.404 |

*Table S3: Mann-Whitney asymptotic rank tests on differences in SWP standard deviation across tasks with Cohen’s d as a measure of effect size: d = T/√N, where N=84 is the total sample size, as well as Bonferroni and Benjamini-Hochberg corrections for average degree thresholds 8, 9, 11, 12.*

| **Average degree** | **Frequency band** | **T** | **p-value** | **Bonferroni**  **p-value** | **Benjamini-Hochberg p-value** | **Effect size** |
| --- | --- | --- | --- | --- | --- | --- |
| **8** | **Delta (1-3 Hz)** | -0.680 | 0.497 | 1 | 0.662 | 0.074 |
|  | **Theta (4-7 Hz)** | -0.215 | 0.830 | 1 | 0.830 | 0.023 |
|  | **Alpha (8-12 Hz)** | 2.800 | 0.005 | 0.020 | 0.020 | 0.306 |
|  | **Beta (13-30 Hz)** | 1.548 | 0.122 | 0.487 | 0.243 | 0.169 |
| **9** | **Delta (1-3 Hz)** | 1.145 | 0.252 | 1 | 0.336 | 0.125 |
|  | **Theta (4-7 Hz)** | 1.306 | 0.192 | 0.766 | 0.336 | 0.143 |
|  | **Alpha (8-12 Hz)** | 2.236 | 0.025 | 0.101 | 0.101 | 0.244 |
|  | **Beta (13-30 Hz)** | 0.877 | 0.381 | 1 | 0.381 | 0.096 |
| **11** | **Delta (1-3 Hz)** | 2.612 | 0.009 | 0.036 | 0.013 | 0.285 |
|  | **Theta (4-7 Hz)** | 2.943 | 0.003 | 0.013 | 0.013 | 0.321 |
|  | **Alpha (8-12 Hz)** | 2.585 | 0.010 | 0.039 | 0.013 | 0.282 |
|  | **Beta (13-30 Hz)** | 1.190 | 0.234 | 0.937 | 0.234 | 0.130 |
| **12** | **Delta (1-3 Hz)** | 1.691 | 0.091 | 0.364 | 0.091 | 0.184 |
|  | **Theta (4-7 Hz)** | 3.328 | 0.001 | 0.004 | 0.004 | 0.363 |
|  | **Alpha (8-12 Hz)** | 1.932 | 0.053 | 0.213 | 0.071 | 0.211 |
|  | **Beta (13-30 Hz)** | 2.827 | 0.005 | 0.019 | 0.009 | 0.308 |
